# Selection and validation of reference genes suitable for gene expression analysis by Reverse Transcription Quantitative real-time PCR in *Acinetobacter baumannii*

**DOI:** 10.1038/s41598-024-51499-5

**Published:** 2024-02-15

**Authors:** Paloma Aparecida Alves de Oliveira, Juliana Baboghlian, Clarissa Orandina Aparecida Ramos, Alquiandra Stefani Ferreira Mançano, Andréia de Melo Porcari, Raquel Girardello, Lúcio Fábio Caldas Ferraz

**Affiliations:** 1 Laboratório de Biologia Molecular de Microrganismos, Universidade São Francisco, Bragança Paulista, SP CEP 12916-900 Brazil; 2 Laboratório Multidisciplinar de Pesquisa, Universidade São Francisco, Bragança Paulista, SP CEP 12916-900 Brazil

**Keywords:** Gene expression, Bacterial genes, Clinical microbiology

## Abstract

*Acinetobacter baumannii* is a Gram-negative bacterium considered an emerging multi-drug-resistant pathogen. Furthermore, this bacterium can survive in extreme environmental conditions, which makes it a frequent cause of nosocomial infection outbreaks. Gene expression analyses by Reverse Transcription Quantitative real-time PCR (RT-qPCR) depend on a reference gene, also called an endogenous gene, which is used to normalize the generated data and thus ensure an accurate analysis with minimal errors. Currently, gene expression analyses in *A. baumannii* are compromised, as there are no reports in the literature describing the identification of validated reference genes for use in RT-qPCR analyses. For this reason, we selected twelve candidate reference genes of *A. baumannii* and assessed their expression profile under different experimental and culture conditions. The expression stability of the candidate genes was evaluated by using statistical algorithms such as BestKeeper, geNorm, NormFinder, Delta C_T_, and RefFinder, in order to identify the most suitable candidate reference genes for RT-qPCR analyses. The statistical analyses indicated *rpoB*, *rpoD*, and *fabD* genes as the most adequate to ensure accurate normalization of RT-qPCR data in *A. baumannii*. The accuracy of the proposed reference genes was validated by using them to normalize the expression of the *ompA* gene, encoding the outer membrane protein A, in *A. baumannii* sensible and resistant to the antibiotic polymyxin. The present work provides suitable reference genes for precise RT-qPCR data normalization on future gene expression studies with *A. baumannii*.

## Introduction

*Acinetobacter baumannii* is an opportunistic pathogen, primarily associated with hospital-acquired infections, and which has proved to be an eminently problematic pathogen globally. *A. baumannii* needs minimal nutritional requirements, which makes it able to survive in extreme environmental conditions and last for long periods on surfaces. Since they are ubiquitous in nature, *Acinetobacter* species are intrinsically resistant to most currently available antibiotics. In addition, *A. baumannii* has a great ability to adapt to the environment, being able to acquire virulence and resistance genes from other pathogens, which makes them even more capable of spreading in hospital environments and infecting vulnerable patients, particularly in intensive care units (ICUs). The alarming spread of *A. baumannii* was even more evident during the COVID-19 pandemic, when multidrug-resistant *A. baumannii* strains have figured among the most common secondary bacterial infections in patients with COVID-19, often with a fatal outcome^1,2^.

Carbapenems remain effective for the treatment of most hospital-acquired critical infections caused by multidrug-resistant Gram-negative bacteria, specially *A. baumannii*. However, the emergence of carbapenem-resistant *Acinetobacter baumannii* (CRAB) over the last decades has become a serious problem of healthcare-associated infections worldwide due to the limited options of antimicrobials for treatment^3–5^. In fact, the WHO has appointed CRAB among the top-priority bacteria that deserve support for the research and development of new effective drugs^6^. Currently, polymyxin antibiotics are recognized as last-resort antibiotics against CRAB treatment, although *A. baumannii* may develop polymyxin resistance and their use is limited due to nephrotoxicity and neurotoxicity^4,5,7–9^.

Among the main virulence factors that allow *A. baumannii* to acquire resistance, the porin OmpA stands out as an outer membrane protein that plays key roles in the pathogenicity of *A. baumannii* by regulating the adhesion, biofilm formation, and the immune response of the host^10–12^. In addition, OmpA plays an important role in resistance to carbapenems^13,14^. Therefore, the expression level of OmpA is shown to be a diagnostic index for *A. baumannii* resistant to antibiotics^15^, either through the reduction of its expression when resistant or by joining the efflux pump mechanism to eject the antibiotic present in the periplasmic space out of the bacterial cell^11,16^. Thus, the characterization of porin OmpA is important to support studies that seek new therapeutic strategies against antibiotic resistance mechanisms.

Considering the current relevance of multidrug resistant strains of *A. baumannii*, it is essential to characterize the virulence genes of this bacterium and how these genes are expressed during the colonization of a host. Different methods of gene expression analysis are available and their choice varies according to budget, laboratory facilities, cost-effectiveness, availability of hardware and software, the purpose of the study, and the number of genes of interest^17^.

Currently, gene expression analyses in *A. baumannii* have been performed by comparative transcriptomic profiling^18–21^, PCR array^22^, reporter vector systems^23^, and mostly by Reverse Transcription Quantitative real-time Polymerase Chain Reaction (RT-qPCR)^7,18,19,24–30^.

Since its first proposal, RT-qPCR has remained the most versatile and ubiquitous gene expression analysis technique due to its practicality and accessibility. The introduction of fluorescent markers not only reduced the risks of contamination but also eliminated the need for post-PCR reaction processing, such as agarose gel electrophoresis^31^. However, a crucial step in RT-qPCR analysis is the normalization of the raw data by an internal control, also called the reference gene or an endogenous gene. The use of a reference gene is essential to achieve reliable results, as in addition to establishing itself as a normalizer for the other genes under study, it will also correct possible errors in pipetting, RNA extraction, or cDNA synthesis^32^. A suitable reference gene for RT-qPCR reactions must have unchanged expression, regardless of the bacterial culture condition^33^. In addition, to be used as an internal control, the candidate reference gene must be experimentally validated, following the Minimum Information for Publication Quantitative Real-Time PCR Experiments (MIQE) guidelines^31^.

Unfortunately, there are no reports of a comprehensive evaluation of experimentally validated reference genes to be used in RT-qPCR in *A. baumannii*. Therefore, we describe here the investigation of twelve candidate genes as potential internal controls in RT-qPCR analyses with *A. baumannii*. The expression pattern of the candidate genes was measured in *A. baumannii* subjected to different experimental conditions, and the stability of their expression were statistically evaluated using Bestkeeper, NormFinder, geNorm, Delta C_T_, and RefFinder programs. Statistical analyzes ranked *rpoB*, *rpoD*, and *fabD* as the most suitable reference genes, and these genes were used to normalize the expression of the *ompA* gene in clinical samples of *A. baumannii* sensitive and resistant to the antibiotic polymyxin. The contribution of the present study was to provide suitable reference genes for precise RT-qPCR data normalization so that future analysis of gene expression on *A. baumannii* be precisely performed.

## Results

### Selection of candidate reference genes, PCR amplification efficiencies, and expression profile under different experimental conditions

In this study, the following 12 genes were selected and evaluated as potential candidate reference genes for RT-qPCR in *A. baumannii*: *16S*, *atpD*, *era*, *fabD*, *ftsZ*, *gapA*, *groEL*, *gyrA*, *proC*, *rho*, *rpoB*, and *rpoD*. The efficiency of PCR amplification of primer pairs for each gene was calculated from standard curves constructed from RT-qPCR reactions performed with serial dilutions of chromosomal DNA from the bacteria. The expression profile of the candidate genes was evaluated from *A. baumannii* subjected to four different culture conditions, namely: (1) cells isolated at different growth phases (lag, mid-log, and stationary phases); (2) cells subjected to pH conditions above and below the ideal for their growth (pH 9.0 and 5.0, respectively); (3) cells submitted to thermal shock at temperatures also above and below the ideal for their growth (42 °C and 28 °C, respectively), and (4) cells grown in solid LB medium. Supplementary Fig. S1 shows the growth patterns of *A. baumannii* grown in LB broth and under stressful cultivation conditions.

Table 1 shows the candidate genes selected in this study, the product they encode, the nucleotide sequences of the primer pairs for each gene, and the amplicon size. The PCR amplification specificity of each primer pair was confirmed by the presence of a single PCR product of the expected size on agarose gel electrophoresis (Supplementary Fig. S2). Furthermore, all 12 candidate genes presented a single peak in the dissociation-curve analyses, indicating the absence of primer-dimers and/or non-specific PCR products (Supplementary Fig. S3).

**Table 1:** Selected candidate reference genes, their corresponding product name, primer sequences (annealing temperature of 60 °C), amplicon size in base pairs (pb), their respective PCR amplification efficiencies and the mean C_T_ values (± standard deviation) assessed in *A. baumannii* cells submitted to various experimental conditions and at different phases of growth.

Genes	Product name	Forward and reverse primers sequences (5’ > 3’)	Amplicon size	R^2^	E (%)	Mean C_T_ ± SD
*16S*	16S ribosomal RNA	TAGTCCATGCCGTAAACGATGT TTGAGTTTTAGTCTTGCGACCG	114 pb	0.998	108	16.03 ± 1.71
*atpD*	ATP synthase subunit beta	AGATCTATGACGCTCTCCAAGTTG GTAGAACCCATTGCGATGGTAC	100 pb	0.996	110	22.58 ± 1.1
*era*	GTPase Era	GATCAATTTTTTAGTTCCAAAGGCGT TTCATGAGGGTAGATTTACCCACA	104 pb	0.991	122	–
*fabD*	ACP S-malonyltransferase	TTGCAGAAGCTTTGGAACAAACT CGTAATTGAGCAACATCGGTAGC	97 pb	0.995	115	23.15 ± 0.49
*ftsZ*	Cell division protein FtsZ	GCTGGTATGGGTGGTGGTAC GACGGCCTTCAAAGTTAAATGG	118 pb	0.986	121	–
*gapA*	Glyceraldehyde-3-Phosphate dehydrogenase A	ACCAAGGCCGTTGGAAAAAC AGAGCGACCGTAAACAGATACG	107 pb	0.993	112	22.85 ± 0.94
*groEL*	Chaperonin GroEL	GTGTTCAACAAATCCGTGCTC TAATTACAGCAACACCGCCTG	105 pb	0.992	102	21.13 ± 0.7
*gyrA*	DNA gyrase subunit A	AACCTGTTCACCGTCGTGTG ATTTACCGATTACGTCCCCAAC	105 pb	0.990	105	23.74 ± 0.8
*proC*	Pyrroline-5-carboxylate reductase	GGTGCAGCTCAAATGGCAATTA GATCAAAAACTTCAAGTGCAGCTTG	106 pb	0.983	105	24.79 ± 0.98
*rho*	Transcription termination Factor Rho	AATTAAAATTGCTGAATTTATGGGC CACCAAAAATTTCTTCGCCAT	113 pb	0.993	104	21.43 ± 0.85
*rpoB*	DNA-directed RNA Polymerase subunit beta	ACGCCTAAAGGTGAAACTCAGTTAA GTACCAGATGGAACACGTAAAGATG	110 pb	0.992	105	22.53 ± 0.59
*rpoD*	RNA polymerase Sigma Factor RpoD	GTTGCTGAAGAAGAAGCTGCTG ACTGTACCCATTTCACGCATGTA	101 pb	0.989	105	22.83 ± 0.36

Product name according to the annotated genome sequence of *Acinetobacter baumannii* ATCC 19606 (GenBank accession number CP045110.1). R^2^: correlation coefficient; E: PCR efficiency (%); S.D.: standard deviation.

Table 1 also displays the calculations for the PCR amplification efficiencies (E) for each pair of primers, which were calculated based on standard curves generated for each gene, and the cycle threshold (C_T_) values. The PCR efficiency of *era* and *ftsZ* genes, of 122% and 121% respectively, were above the acceptable range and, therefore, were excluded from the subsequent analyses. The PCR efficiency of the other genes ranged from 102% for *groEL* to 115% for *fabD*, which were within the acceptable range.

The C_T_ values ranged from 16.03 to 24.79, with most genes having average C_T_ values of 22.06. The *16S* ribosomal gene presented the lowest C_T_ value (16.03 ± 1.71, mean C_T_ ± standard deviation) and, therefore, was the most abundantly expressed gene across all samples. On the other hand, the *proC* gene was considered the least expressed gene since it had the highest C_T_ (24.79 ± 0.98, C_T_ mean ± standard deviation). The *16S* and *atpD* genes presented the largest standard deviation of the mean C_T_ values (standard deviation of 1.71 and 1.1, respectively), whereas *rpoD* and *fabD* genes showed the least variation on the standard variation (0.36 and 0.49, respectively).

### Expression stability analyses of candidate reference genes

The stability of transcription levels of candidate genes under the different experimental conditions was evaluated using the statistical algorithms BestKeeper, geNorm, NormFinder, Delta C_T_, and RefFinder; the latter program integrates all four algorithms to elaborate a comprehensive analysis. Table 2 summarizes the ranking of the genes with the most stable expression across all experimental conditions tested and calculated by all five statistical programs.

**Table 2:** Expression stability ranking of the candidate reference genes according to BestKeeper, NormFinder, geNorm, Delta C_T_ and RefFinder analyses.

Ranking	BestKeeper		NormFinder		geNorm		Delta C_T_		RefFinder	
	Gene	SD value	Gene	Stability value	Gene	*M* value	Gene	Average of SD	Gene	Geomean of ranking values
1	*fabD*	0.484	*rpoB*	0.315	*rpoB/rpoD*	0.519	*rpoB*	0.923	*rpoB*	1.41
2	*rpoD*	0.522	*rpoD*	0.430	*rpoB/rpoD*	0.519	*fabD*	0.943	*rpoD*	1.86
3	*groEL*	0.55	*fabD*	0.448	*fabD*	0.559	*rpoD*	0.968	*fabD*	2.06
4	*rpoB*	0.60	*gyrA*	0.465	*gyrA*	0.671	*gyrA*	0.971	*gyrA*	4.23
5	*gyrA*	0.645	*gapA*	0.751	*gapA*	0.738	*gapA*	1.092	*gapA*	5.44
6	*rho*	0.748	*proC*	0.804	*proC*	0.787	*proC*	1.138	*proC*	6.64
7	*gapA*	0.814	*rho*	0.812	*rho*	0.857	*rho*	1.169	*rho*	6.74
8	*atpD*	0.898	*atpD*	1.016	*atpD*	0.928	*atpD*	1.284	*groEL*	6.84
9	*proC*	0.986	*groEL*	1.069	*groEL*	0.996	*groEL*	1.327	*atpD*	8.0
10	*16S*	1.534	*16S*	1.711	*16S*	1.166	*16S*	1.845	*16S*	10.0

BestKeeper ranks the most stable gene based on the correlation coefficient (*r*), the coefficient of variation of the C_Ts_ (CV values), and the standard deviation (SD values). Genes with the lowest SD values and highest coefficient of correlation are considered the most stable. BestKeeper ranked *fabD* and *rpoD* with the lowest SD values (0.484 and 0.522, respectively) and, therefore, the genes with the most stable expression under the tested experimental conditions (Table 2). According to BestKeeper, genes with SD values greater than 1 are considered inappropriate as reference genes. Among all tested genes, only *16S* presented SD value greater than 1 (1.534). This result corroborates the result presented in Table 1, which showed *16S* as the gene with the greatest variation in the C_T_ number among all the genes analyzed.

geNorm determines the expression stability of each gene by calculating an expression stability M value. The program recommends a cut-off of M < 1.5: the lower the value, more stable is the expression of the gene. geNorm indicated the *rpoB*, *rpoD*, and *fabD* genes with the lowest M values (0.519, 0.519, and 0.556, respectively) and, therefore, with the most stable expression (Table 2). On the other hand, the program determined *16S* with the highest M value (1.166) and, thus, the least stable gene.

**Figure 1:**
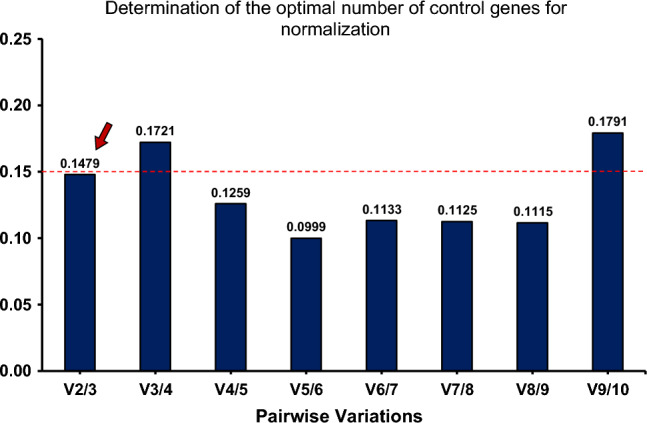
geNorm analysis indicates the optimal number of reference genes for normalization of RT-qPCR data. geNorm calculates the pairwise variation (V_n_/V_n+1_, V value) to determine the minimum number of reference genes required for accurate normalization. The program suggests a cut-off threshold of 0.15 (indicated by the red dashed line), below which the inclusion of an additional reference gene is not necessary. The V_2/3_ value of 0.1479 (red arrow) indicates that the inclusion of the third most stable reference gene (*fabD*) would have no significant contribution to the accuracy of the data normalization.

geNorm also employs pairwise variation (V_n_/V_n+1_) analyses to generate a V value that indicates the optimal number of reference genes needed for the reliable normalization of RT-qPCR data. The program recommends a V value threshold of 0.15, below which the addition of more reference genes is not required. As displayed in Fig. 1, the pairwise variation of V_2/3_, considering the two most stable genes (*rpoB* and *rpoD*), was below the cut-off of 0.15 (0.1479), indicating that the addition of a third gene is not necessary for an accurate normalization.

NormFinder combines intra- and inter-group variations to calculate a stability (S) value: genes with the lowest S values present the most stable expression. As shown in Table 2, NormFinder indicated *rpoB*, *rpoD*, and *fabD* as the top ranked genes with S values of 0.315, 0.430, and 0.448, respectively. On the other hand, NormFinder indicated *16S* (S value of 1.711) and *groEL* (S value of 1.069) with the highest S values and, therefore, with the least stable expression. The stability ranking performed by the Delta C_T_ method was similar to the one generated by NormFinder, indicating *rpoB*, *fabD*, and *rpoD* as the top ranked genes with the lowest average of the standard deviation (SD of 0.923, 0.943, and 0.968, respectively).

Finally, Table 2 also shows the ranking of the most stable genes generated by RefFinder. This is a web-based program that generates a comprehensive ranking based on the geometric mean of the results obtained by the other statistical analysis softwares (geNorm, NormFinder, BestKeeper, and Delta C_T_ methods). This comprehensive ranking generated by RefFinder indicated *rpoB*, *rpoD*, and *fabD* as the three top-ranked genes with the lowest geometric mean (geomean) of ranking values (1.41, 1.86, and 2.06, respectively).

The proposed reference genes are highly conserved among different strains of *A. baumannii* and other species of the genus *Acinetobacter*. The primer sequences for each gene on *A. baumannii* strains AB736 and AB5075-UW and on other species of the genus *Acinetobacter*, such as *A. calcoaceticus, A. haemolyticus*, *A. johnsonii, A. junii*, *A. lwoffii*, *A. nosocomialis, A. pittii*, and *A. schindleri*, are displayed on Supplementary Tables S1–S11.

### Validation of selected reference genes by expression analyses of *ompA* gene

To evaluate the reliability of the selected reference genes, the three genes with the most stable expression in this study—*rpoB*, *rpoD*, and *fabD*—were used to normalize the expression of *ompA* gene in a clinical isolate of *A. baumannii* sensitive to the antibiotic polymyxin B and after acquiring resistant to this antibiotic. To compare the normalization using the most stable genes with the least stable, the *16S* gene was also used to normalize the RT-qPCR data, since it is commonly used as a reference gene for *A. baumannii* and presented the least stable expression in the present study.

**Figure 2:**
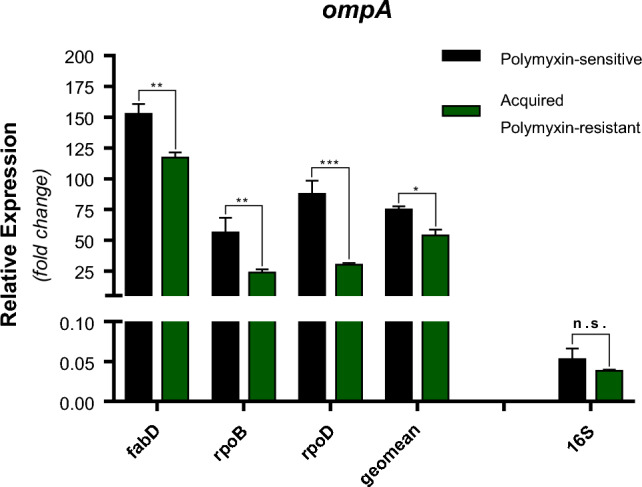
Relative expression of the *ompA* gene in a clinical isolate of *A. baumannii* sensitive to polymyxin and after having acquired resistance to this antibiotic. The expression data were normalized by the three most stable genes—*fabD*, *rpoB*, and *rpoD*—individually and with a geometric mean across the three genes (geomean). As a comparison, *ompA* expression was normalized with *16S*, the least stable gene. *p < 0.05, **p ≤ 0.01, ***p ≤ 0.001, and n.s.: not significant (*p* ≥ 0.05).

As shown in Fig. 2, the normalization of the RT-qPCR data using the three most stable reference genes alone or as a geometric mean revealed statistically significant down-regulation of *ompA* gene at the isolate resistant to polymyxin when compared to the isolate sensitive to the antibiotic. On the other hand, normalization with the *16S* gene rendered results similar to the one obtained with the most stable genes, but the results were sub-estimated and not statistically significant.

## Discussion

*A. baumannii* is currently considered a pathogenic bacterium of clinical relevance, mainly due to the emergence of strains resistant to multiple drugs and associated with infections related to health care^34,35^. In this scenario, genetic tools that allow the study of genes that encode virulence factors and confer resistance to antimicrobials is of paramount importance for a better understanding of the pathogenicity of this bacterium^36^.

The RT-qPCR technique is considered the gold standard method in gene expression analysis^37^. One of the most critical steps in this technique is the use of reliable reference genes, which act as internal controls of the RTq-PCR reactions and are fundamental to ensure a proper normalization of the obtained results. The choice of a reference gene for RT-qPCR data normalizations should involve careful selection, as an adequate endogenous gene should preferably have a stable expression, regardless of the conditions in which the bacterium is subjected^31,38^. Most of the time, the genes that normally show a stable expression pattern are the constitutive genes, which encode proteins of the bacteria's basal metabolism^33^.

Unfortunately, there is no description in the literature of properly validated reference genes for RT-qPCR for *A. baumannii*, despite the clinical significance of this bacterium. Although their stable expressions have not been experimentally validated, several genes have been used in RT-qPCR analysis in *A. baumannii*, such as *groEL*^22^, *gyrB*^18^, *gyrA*^30^, *recA*^22^, *rpoB*^22^, *rpoD*^29^, and the ribosomal RNA coding genes *50S*^39^ and mostly *16S*^7,19,24–28^. However, the use of a gene encoding a ribosomal RNA as an internal control in RT-qPCR reactions may be challenging, as its high expression hinders its use in mRNA transcript normalizations^33^.

In response to the lack of suitable reference genes for gene expression analysis in *A. baumannii*, a systematic approach was performed in this study to identify and validate ideal reference genes to be used in RT-qPCR analyses in *A. baumannii*. Twelve candidate reference genes (*16S*, *atpD*, *era*, *fabD*, *gapA*, *groEL*, *gyrA*, *proC*, *rho*, *rpoB*, and *rpoD*) commonly used in other bacterial species were selected and evaluated, some of them already used in *A. baumannii* despite of not having their stable expressions validated. The set of primers designed for the candidate genes yielded amplicon sizes ranging from 97 to 118 bp, which is within the range recommended for SYBR Green-based assays^40,41^.

The expression profile of the selected genes was evaluated in *A. baumannii* cells submitted to different experimental conditions and then the stability of the expression of the candidate genes was checked using the statistical algorithms Bestkeeper, geNorm, NormFinder, Delta C_T_, and RefFinder. Except for the Bestkeeper analysis, all other statistical algorithms (geNorm, NormFinder, Delta C_T_, and RefFinder) recommended *rpoB*, *rpoD*, and *fabD* as the top three most stable reference genes across all the experimental conditions, with only a slight variation in the ranking order by the Delta C_T_ method. BestKeeper's analysis presented the greatest difference in the ranking of the most stable candidate genes. Although Bestkeeper also ranked *rpoD* and *fabD* among the three most stable genes, the program ranked *groEL* as the third and *rpoB* as the fourth most stable genes. However, *groEL* was not considered a stable gene by the other statistical programs. On the contrary, most algorithms ranked the *groEL* gene among the genes with the least stable expression. Thus, we suggest that the *groEL* gene be disregarded as a stable gene for RT-qPCR data normalizations in *A. baumannii*. Unanimously, all five statistical programs indicated the *16S* gene as the one with the least stable expression and, therefore, the least suitable for normalizations of RT-qPCR data in *A. baumannii*.

Based on the evaluation by all statistical programs, this study suggests the *rpoB*, *rpoD*, and *fabD* genes as the most suitable reference genes for normalizing RT-qPCR data in *A. baumannii*. Congruent with our study, these genes were also indicated as internal controls for analysis of RT-qPCR data in other bacteria, such as in *Pseudomonas brassicacearum* GS20^42^, *Staphylococcus aureus*^43,44^, *Saccharopolyspora spinosa*^45^, and also for *Aeromonas salmonicida*^46^.

In order to test the legitimacy of the proposed genes as normalizers of RT-qPCR reactions in *A. baumannii*, we used the expression of *fabD*, *rpoB*, and *rpoD* to normalize the expression of the *ompA* gene in a strain of *A. baumannii* sensitive to the polymyxin B antibiotic and after the same strain acquires resistance to this antibiotic. As a comparison, we also used the least stable gene expression, *16S*, as a normalizer of *ompA* expression. We chose to normalize *ompA* expression in these conditions because polymyxin B is the main therapeutic option available to treat infections caused by *A. baumannii* strains resistant to multiple drugs. The *ompA* gene encodes the outer membrane protein OmpA, which is considered not only a virulence factor, as it acts as an adhesin in the bacteria's adhesion processes, but it is also of great importance for the genus *Acinetobacter* because it is involved in resistance to antibiotics of the class of beta-lactams^13^.

In the present study, the normalization of *ompA* expression using the three recommended genes (*rpoB*, *rpoD*, and *fabD*) generated the same expression pattern, either performing the normalization with the genes individually or using a geometric mean between them. In contrast, normalization with the *16S* gene generated underestimated and statistically non-significant data, despite resulting in an expression pattern similar to that obtained with the recommended reference genes. Therefore, despite the *16S* gene being used as a reference gene for the bacteria *A. baumannii* in some studies, caution should be taken when using this gene on RT-qPCR normalizations in *A. baumannii*.

In a study that evaluated the mechanisms of antimicrobial resistance in clinical isolates of *A. baumannii* initially susceptible to polymyxin B and after acquiring resistance to this antibiotic, the authors showed changes in the surface of *A. baumannii* strains submitted to cultivation in the presence of polymyxin B, in addition to changes in the expression of genes that encode outer membrane proteins, such as porins and efflux pumps^7^. The data obtained in the present study show that while the expression of the *ompA* gene is induced in polymyxin-sensitive *A. baumannii*, the expression of the gene is reduced when the same strain becomes resistant to polymyxin. This result suggests that resistance to polymyxin acquired by bacteria does not seem to involve the expression of the outer membrane protein OmpA. Interestingly, this result goes against a study that showed that the resistance of *A. baumannii* to polymyxin is due to the presence of OmpA in the bacteria, as the knockout of the *ompA* gene makes the bacteria more sensitive to the antibiotic^13,14^. However, this reduction in polymyxin resistance is much more likely due to the loss of cell wall integrity in a mutant strain lacking the OmpA protein.

A limitation of the present study is that, except for the static culture in a solid LB medium, the expression stability of the proposed reference genes was defined from cultivation and experimental conditions that involved the planktonic mode of growth. It is plausible to state that these genes may not be equally suitable under other experimental conditions. For this reason, it is strongly advised that the expression stability of the proposed reference genes be checked in any physiological or experimental condition to be investigated, to guarantee the reliability of the normalization of RT-qPCR data with these genes. For instance, biofilm formation has recently become a major research field in pathogenicity studies with *A. baumannii*^18,26,47,48^. Before using the proposed endogenous genes in gene expression analysis during biofilm growth by *A. baumannii*, it is advisable to test the stability of their expression in this specific condition. Likewise, although the proposed genes are highly homologous between different strains of *A. baumannii* and even between other species of the genus *Acinetobacter*, it is worth testing the expression stability of the proposed reference genes and their suitability on RT-qPCR data normalization in a given strain or *Acinetobacter* species under study. As there is no ideal reference gene with stable expression regardless of physiological state and experimental conditions, the stable expression of any potential reference gene must be validated under all physiological conditions or experimental treatment under study before its use in RT-qPCR normalizations^33^.

In summary, a reliable normalizing of RT-qPCR data relies on the use of stable reference genes, which demands testing the candidate reference genes at the most different experimental conditions. In the present study, *rpoB*, *rpoD*, and *fabD* were recommended as reliable reference genes for RT-qPCR data normalization in *A. baumannii* under the chosen experimental conditions. It is advisable that the expression stability of the proposed reference genes be assessed in a given experimental treatment under study, prior to their use on the normalization of gene expression by RT-qPCR analysis in this bacterium. To our knowledge, this is the first study aimed to identify reference genes for RT-qPCR analysis in *A. baumannii*.

## Methods

### Selection of candidate reference genes for *A. baumannii*

The selection of endogenous candidate genes was performed on the annotated genome of *A. baumannii* ATCC 19606 (NCBI reference sequence NZ_CP045110.1, available at https://www.ncbi.nlm.nih.gov/nuccore/NZ_CP045110.1?report=gbwithparts&log$=seqview). Special attention must be given to the choice of candidate genes to be evaluated, which must involve a rigorous gene selection procedure and taking into account the specific conditions under which the study will be conducted^49,50^. The criterion used in the present study for selecting candidate genes was to choose internal controls commonly used in other bacteria in the most diverse cultivation and experimental conditions as described in the literature. Among the selected genes are *16S* (16S ribosomal RNA)^7,19,24–28,51^, *atpD* (ATP synthase β subunit)^52^, *era* (era GTPase)^53^, *fabD* (ACP S-malonyltransferase)^54^, *ftsZ* (cell division protein FtsZ)^42,55,56^, *gapA* (glyceraldehyde-3-phosphate dehydrogenase A)^57^, *groEL* (GroEL chaperonin)^22,58,59^, *gyrA* (DNA gyrase subunit A)^30,42,60^, *proC* (pyrroline-5-carboxylate reductase)^33,61^, *rho* (transcription termination factor Rho)^33,42,45^, *rpoB* (β subunit of RNA polymerase)^22,44,45,62^ and *rpoD* (RNA polymerase Sigma factor RpoD)^33,42,63^. In order to minimize the chance of coregulation of the selected genes, the candidate genes were selected in order to belong to different functional categories such as cellular metabolism (*atpD*, *era*, *fabD*, *gapA*, and *proC*), protein synthesis (*groEL* and *16S*), cell division (*ftsZ*) and DNA metabolism (*gyrA*, *rho*, *rpoB*, and *rpoD*).

BLAST multiple alignments were performed to confer the homology of the proposed genes from *A. baumannii* ATCC 19606 with the homologous genes from other strains of *A. baumannii* and other species of the genus *Acinetobacter*.

### Primers design and PCR amplification efficiencies

Pairs of primers specific for each selected gene were designed with the web-based program Primer3 v. 0.4.0^64^, from the nucleotide sequence of the coding region of each gene. Primer pairs were designed in order to present an annealing temperature of 60 °C, GC content of 40–60%, and yield product size of 95–120 bp.

Primers were initially tested by conventional PCR, using genomic DNA from *A. baumannii* ATCC 19606, extracted using the *Wizard® Genomic DNA Purification* kit (Promega), and the *Platinum™ PCR SuperMix* kit (Invitrogen™), following the instructions of the manufacturers. The amplicons were analyzed by agarose gel electrophoresis to confirm the specificity of the PCR amplification. The images were captured with a digital Gel Doc™ XR photodocumentation system (Biorad) and analyzed with the Image Lab™ software version 5.0 (Biorad).

To determine the PCR amplification efficiency and the regression coefficient (R^2^) of each pair of primers, standard curves for each selected candidate gene were constructed from real-time PCR reactions performed with increasing concentrations of genomic DNA extracted from the bacteria. The reactions were performed in a 7300 Real-Time PCR System equipment (Applied Biosystems™) with the *Platinum™ SYBR™ Green qPCR SuperMix-UDG w/ROX* kit (Invitrogen™), following the manufacturer's instructions. At the end, an additional dissociation step was performed (40 cycles with a decrease of 1 °C every 15 s, starting at 95 °C) to generate a dissociation (melting) curve. The analysis of this curve made it possible to verify the specificity of the reaction, that is, whether there were nonspecific bands and/or primer-dimers formation. The analysis of the generated data was performed using the 7300 Real-Time PCR System Sequence Detection software v1.4.1 (Applied Biosystems™).

Standard curves were constructed for each candidate gene by plotting the mean C_T_ numbers versus the logarithmic amount of the serially diluted template DNA. The formula E (%) = [10(− 1/slope) − 1] × 100 was used to calculate the PCR amplification efficiency (E) of each pair of primers, and the efficiency of each gene was considered in the subsequent statistical analyses. In this process, the *era* and *ftsZ* genes were eliminated on further analyses, as they presented a slope value outside the optimal range.

### Bacterial strain and culture conditions

*A. baumannii* strain ATCC 19606 was used in this study. Bacteria were cultured in LB broth at 37 °C and under 250 rpm of agitation. Bacterial growth was monitored by reading the optical density in a spectrophotometer at a wavelength of 600  nanometers (D.O._600 nm_), using the GeneQuant spectrophotometer (GE Healthcare).

The expression of the selected genes was measured from *A. baumannii* cultivated under eight different experimental conditions, namely: cultivation of the bacteria in LB broth until the growth phases lag (D.O._600 nm_ = 0.2), exponential (D.O._600 nm_ = 0.6), and stationary (D.O._600 nm_ = 1.0); growth of the bacterial cells until the exponential phase (D.O._600 nm_ of around 0.5) under the thermal stress of 28 °C and 42 °C; growth of the bacterial cells until the exponential phase (D.O._600 nm_ of around 0.5) under the pH stress of 5.0 and 9.0, and cultivation of *A. baumannii* in solid LB medium.

To validate the reference genes recommended by the statistical programs, the expression pattern of the *ompA* gene was analyzed in an *A. baumannii* strain sensitive to the antibiotic polymyxin B isolated from bloodstream infection. The expression pattern of *ompA* was compared in the strain initially susceptible to polymixin B and after it had acquired resistance to this antibiotic. Antibiotic resistance was induced by culturing the clinical isolate in increasing concentrations of polymyxin B sulfate (Sigma-Aldrich) on 10 consecutive days, starting with a concentration of 0.125 µg/mL. Subcultures were carried out in solid LB medium until a minimum inhibitory concentration (MIC) of 4 µg/mL was obtained, which corresponds to polymyxin B resistance according to the Clinical and Laboratory Standards Institute (CLSI) guidelines^65^. The expression of the *ompA* gene was investigated in polymyxin B sensitive and resistant strains grown in TSB medium (Himedia) up to the exponential phase of growth. The resistant strain was grown in the presence of 4 µg/mL of polymyxin B in order to maintain resistance.

Bacterial cells cultured under the abovementioned conditions were harvested by centrifugation, and the cell pellets were kept in *RNAprotect®* *Bacteria Reagent* (Qiagen) for RNA stabilization until the total RNA extraction procedures.

### RNA extraction, cDNA synthesis and real-time PCR reactions

Total bacterial RNA and cDNA synthesis were performed following protocols published elsewhere^66,67^. Total RNA was extracted from the *A. baumannii* cells cultured under the abovementioned conditions by using the *TRIzol™ Plus RNA Purification kit and Phasemaker™ Tubes Complete System* (Invitrogen™), following the manufacturer’s instructions. To ensure a DNA-free total RNA, on-column DNase treatments were performed using *PureLink™ DNase* (Invitrogen™), according to the manufacturer’s recommendations. The integrity of the extracted RNA was analyzed by agarose gel electrophoresis and its purity and concentration were calculated through the optical density of the samples at 260 and 280 nm using the NanoDrop 2000 spectrophotometer (ThermoFisher).

For the cDNA synthesis, 1 μg of total RNA was reverse transcribed using the *High-Capacity cDNA Reverse Transcription* kit (Applied Biosystems™), as recommended by the manufacturer’s protocol. The real-time PCR reactions were conducted with cDNA samples diluted with nuclease-free water to a final concentration of 100 ηg/uL. Reactions were done in triplicates using the *Platinum™ SYBR™ Green qPCR SuperMix-UDG w/ROX* kit on the 7300 Real-Time PCR System equipment, following the manufacturer’s recommendations. The data obtained were analyzed by the 7300 Real-Time PCR System Sequence Detection software v1.4.1, which generated the raw cycle threshold (C_T_) values for each reaction. The means of the C_T_ values of each candidate reference gene were used on the subsequent statistical analyses to determine the expression stability of the candidate genes.

### Statistical analyses of the expression stability of the reference genes

To identify reference genes with stable and reliable expression for normalization of RT-qPCR data, the expression stability of the candidate genes was evaluated on *A. baumannii* cultured under the abovementioned conditions, using the statistical algorithms BestKeeper, geNorm, NormFinder, Delta C_T_, and RefFinder.

Bestkeeper uses raw C_T_ values to calculate the standard deviation (SD) of the C_T_ values (SD values) and the coefficient of variation (CV [% C_T_])^68^. Reference genes with the lowest SD values are considered the most stable genes, and genes with SD values greater than 1 are considered inconsistent by Bestkeeper.

To evaluate the expression stability of candidate genes, geNorm requires relative data which is obtained by transforming the raw C_T_ values into 2^−ΔCT^ values where ΔC_T_ is the difference between each C_T_ value and the lowest C_T_ value across all samples. geNorm ranks the most stable genes by calculation an expression stability value *M*, which represents the average pairwise variation of a specific reference gene with all other genes tested^49^. Following a stringent analysis^33^, *M* value ≤ 1.0 indicates the most stable expression gene and suitable for RT-qPCR data normalization. geNorm also indicates the optimal number of reference genes for accurate RT-qPCR normalizations by calculating the pairwise variation (V_n_/V_n+1_) between sequential normalization factors (NF_n_/NF_n+1_) across all experimental conditions. The program recommends a cut-off threshold of 0.15, bellow each the inclusion of additional reference genes is not necessary to improve the normalization accuracy^49^.

NormFinder also utilizes relative data calculated from the raw C_T_ values to identify the genes with the most stable expression. The program estimates the intra- and inter-group expression variations for a given set of experiments to produce a Stability value for each candidate gene^50^. Genes with the smallest Stability value are ranked the most stable according to NormFinder.

Delta C_T_ method compares the relative expression of “pairs of genes” within each sample to identify suitable reference genes for accurate RT-qPCR normalization^69^. According to the method, the smaller the average of the standard deviations (Average of SD), the smaller the variability in gene expression.

Finally, the expression stability of the candidate reference genes was also analyzed by RefFinder, a web-based program that integrates the abovementioned algorithms (BestKeepr, geNorm, NormFinder, and the Delta C_T_ method)^70^. By using raw C_T_ values, RefFinder generates a comprehensive ranking based on the geometric mean of the ranking values calculate by the other algorithms.

### Validation of the recommended reference genes

To validate the recommended reference genes, the expression of *ompA* on polymyxin- resistant and -sensitive *A. baumannii* strains were normalized using the three most stable reference genes—*rpoB*, *rpoD*, and *fabD*—individually and as a geometric mean of the three genes. Data were also normalized using the *16S*, since this gene was considered the least stable and it is commonly used as internal control in *A. baumannii* RT-qPCR assays. The relative expression levels of the *ompA* gene were calculated using the comparative critical threshold 2^−ΔΔCT^ method^71^. The differences on the expression levels were evaluated by Student’s t test with correction for multiple tests using the GraphPad Prism 7.00 software. Differences were considered statistically significant with p-values ≤ 0.05.

### Supplementary Information

Supplementary Information.

## Data Availability

All data supporting the findings of this study are included within the published article and in its Supplementary Material files.
