# Supplementary material for: Selection and validation of reference genes suitable for gene expression analysis by Reverse Transcription Quantitative real-time PCR in Acinetobacter baumannii
Source: Sci Rep. 2024 Feb 15;14:3830. doi: 10.1038/s41598-024-51499-5 (PMC10869792; doi:10.1038/s41598-024-51499-5)
Supplement: Supplementary file 1 — Supplementary Information. [file 41598_2024_51499_MOESM1_ESM.pdf]

SUPPLEMENTARY INFORMATION

Selection and validation of reference genes suitable for gene expression analysis by Reverse Transcription Quantitative Real-Time PCR in *Acinetobacter baumannii*

Paloma Aparecida Alves de Oliveira[1], Juliana Baboghlian[1], Clarissa Orandina Aparecida Ramos[1], Alquiandra Stefani Ferreira Mançano[1], Andréia Porcari[2], Raquel Girardello[1], Lúcio Fábio Caldas Ferraz[1]

[1]Laboratório de Biologia Molecular de Microrganismos, Universidade São Francisco, Bragança Paulista, SP, CEP 12916-900, Brazil

[2]Laboratório Multidisciplinar de Pesquisa, Universidade São Francisco, Bragança Paulista, SP, CEP 12916-900, Brazil

[*]Corresponding author: lucio.ferraz@usf.edu.br

SUPPLEMENTARY FIGURES

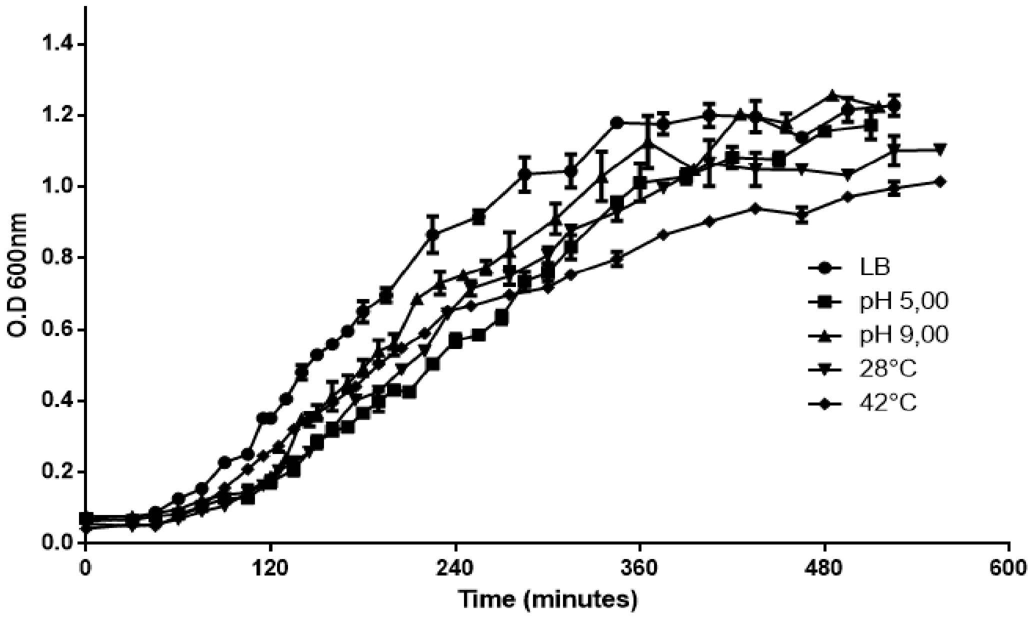

FIGURE S1. Growth curves of *A. baumannii* cultured in LB broth and under stressful cultivation conditions. Cells submitted to the thermal and pH stress have slightly longer growth in the lag phase when compared to growth in LB broth, which led to a slight delay in entering the logarithmic (log) phase. Despite this, *A. baumannii* cells exhibit a similar growth rate at the log phase under the tested conditions.

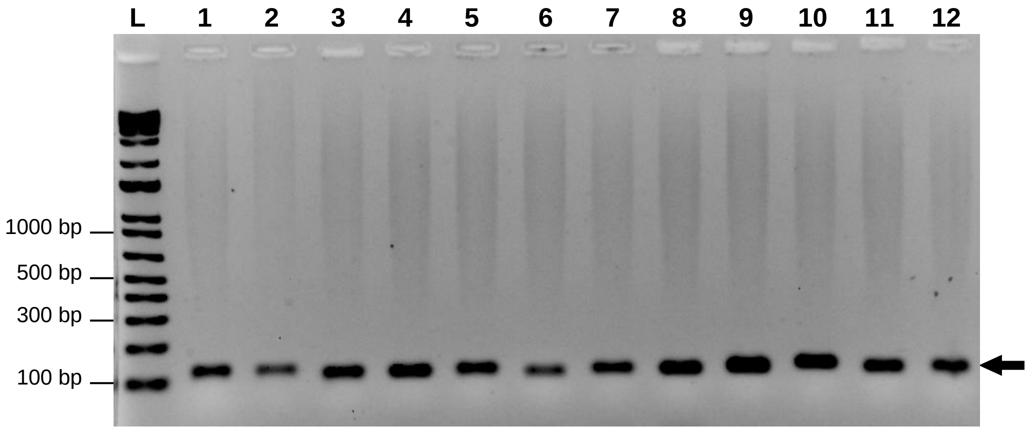

FIGURE S2. Electrophoresis on 1.5% agarose gel reveals single PCR products of expected sizes (arrow) and confirms the specificity of the pairs of primers designed for each gene. **L**, 1 Kb Plus DNA ladder (Thermo Fisher Scientific). **1.** *16S*; **2.** *rpoB*; **3.** *gyrA*; **4.** *atpD*; **5.** *proC*; **6.** *fabD*; **7.** *era*; **8.** *rpoD*; **9.** *gapA*; **10.** *ftsZ*; **11.** *groEL*; **12.** *rho.*

The original uncropped/unprocessed version of the figure is shown below.

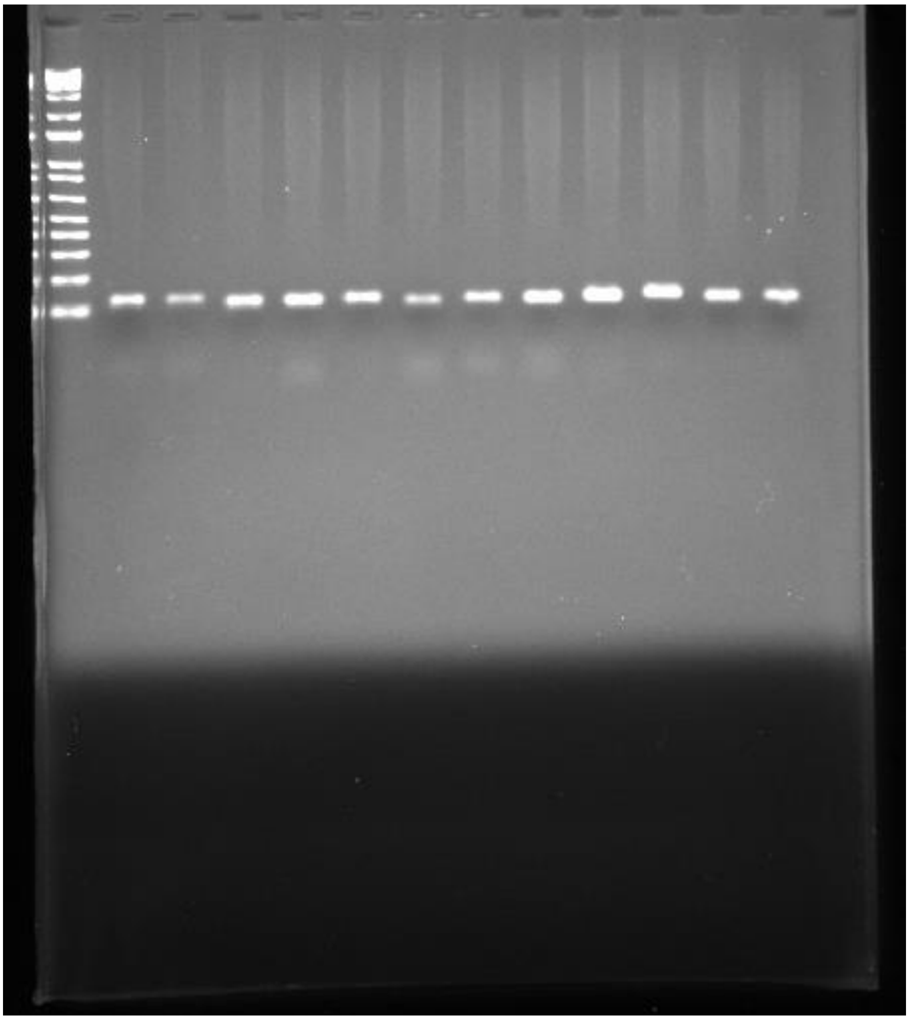

16S

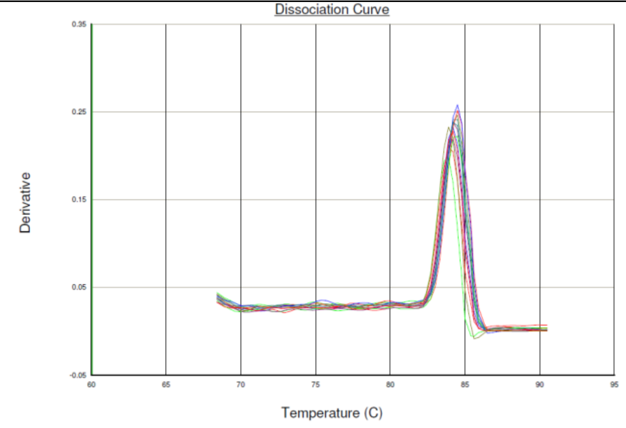

atpD

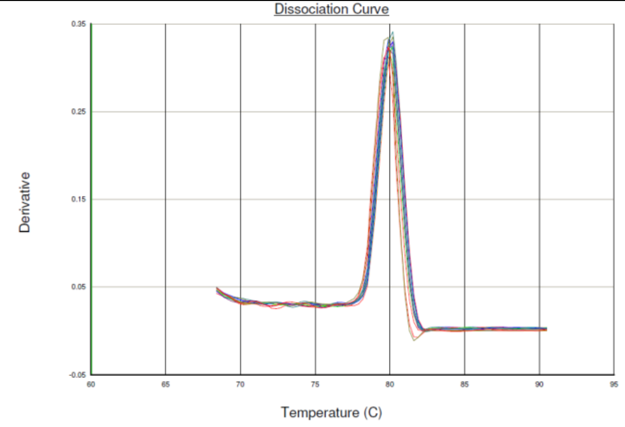

era

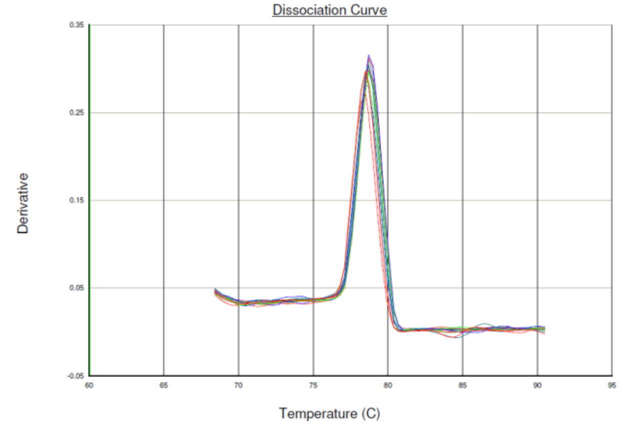

fabD

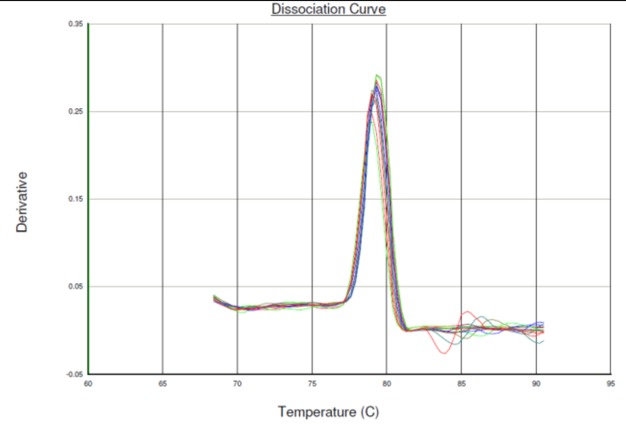

ftsZ

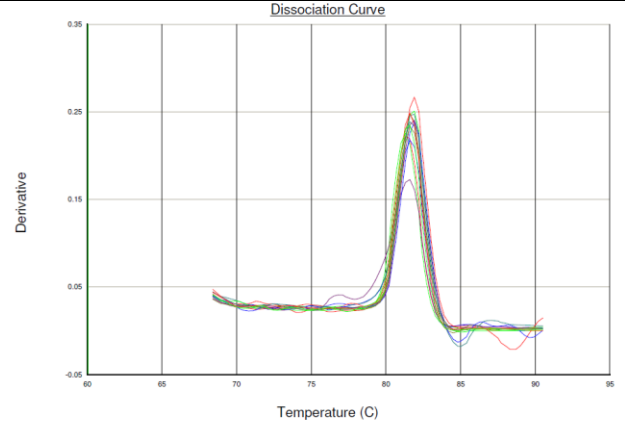

gapA

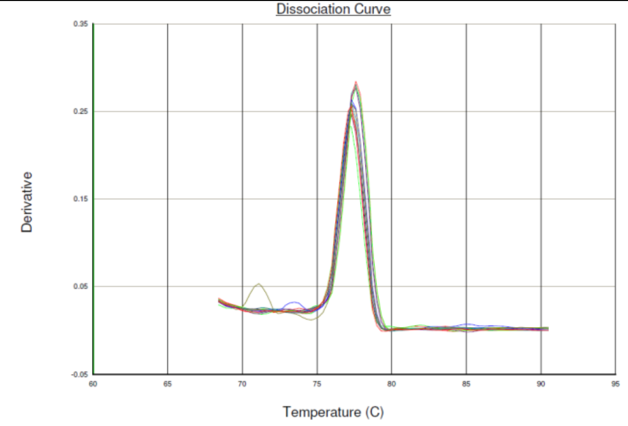

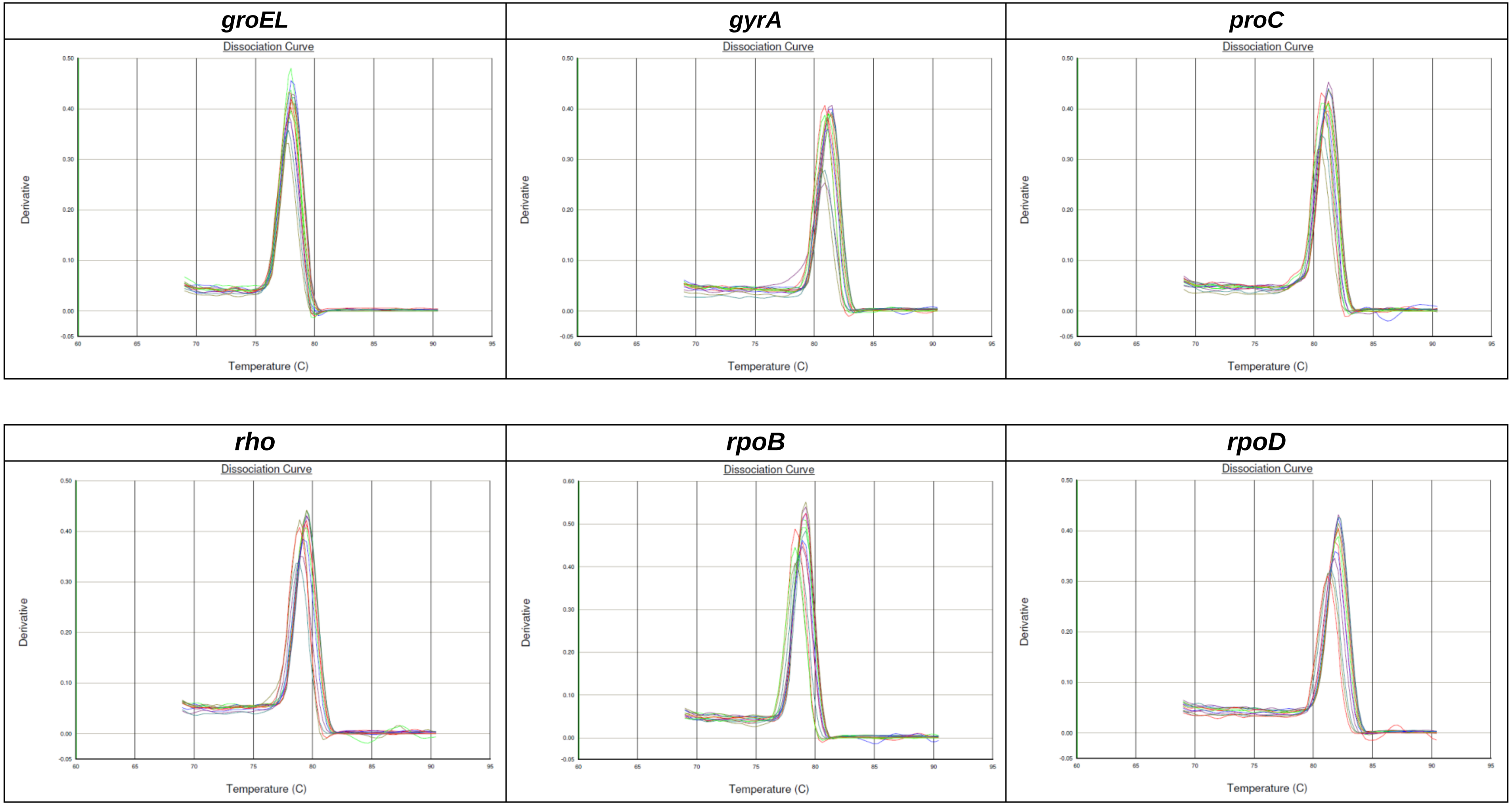

FIGURE S3. Dissociation-curve analyses confirm only single peaks and the absence of primer-dimers formation and nonspecific amplification.

SUPPLEMENTARY TABLES

TABLE S1. Primer sequences of the proposed reference genes (and corresponding locus tag) for *Acinetobacter baumannii* strain AB736 (GenBank: CP015121.1).

Gene	Locus tag	Forward (F) and Reverse (R) primer sequences (5' > 3')	Amplicon size
fabD	A4U85_14830	F- TTGCAGAAGCTTTGGAACAAACT<br>R- CGTAATTGAGCAACATCGGTAGC	97 bp
rpoB	A4U85_17145	F- ACGCCTAAAGGTGAAACTCAGTTAA<br>R- GTACCAGATGGAACACGTAAAGATG	110 bp
rpoD	A4U85_03630	F- GTTGCTGAAGAAGAAGCTGCTG<br>R- ACTGTACCCATTTCACGCATGTA	101 bp

TABLE S2. Primer sequences of the proposed reference genes (and corresponding locus tag) for *Acinetobacter baumannii* strain AB5075-UW (NCBI Reference Sequence: NZ_CP008706.1).

Gene	Locus tag	Forward (F) and Reverse (R) primer sequences (5' > 3')	Amplicon size
fabD	ABUW_RS15095	F- TTGCAGAAGCTTTGGAACAAACT<br>R- CGTAATTGAGCAACATCGGTAGC	97 bp
rpoB	ABUW_RS17475	F- ACGCCTAAAGGTGAAACTCAGTTAA<br>R- GTACCAGATGGAACACGTAAAGATG	110 bp
rpoD	ABUW_RS04230	F- GTTGCTGAAGAAGAAGCTGCTG<br>R- ACTGTACCCATTTCACGCATGTA	101 bp

TABLE S3. Primer sequences of the proposed reference genes (and corresponding locus tag) for *Acinetobacter calcoaceticus* strain 2117 (NCBI Reference Sequence: NZ_LS999521). Nucleotides different from the primers of the *A. baumannii* ATCC 19606 are in red, bold and underlined.

Gene	Locus tag	Forward (F) and Reverse (R) primer sequences (5' > 3')	Amplicon size
fabD	AC2117_RS14710	F- TTGCAGAAGCTTTGGAACAAACT<br>R- CG**C**AATTGAGCAACATCGGTAGC	97 bp
rpoB	AC2117_RS17315	F- ACGCCTAAAGGTGAAACTCAGTTAA<br>R- GTACCAGATGGAACACGTAAAGATG	110 bp
rpoD	AC2117_RS03890	F- GTTGCTGAAGAAGAAGCTGCTG<br>R- ACTGTACCCATTTCACGCATGTA	101 bp

TABLE S4. Primer sequences of the proposed reference genes (and corresponding locus tag) for *Acinetobacter calcoaceticus* strain CA16 (NCBI Reference Sequence: NZ_CP020000.1). Nucleotides different from the primers of the *A. baumannii* ATCC 19606 are in red, bold and underlined.

Gene	Locus tag	Forward (F) and Reverse (R) primer sequences (5' > 3')	Amplicon size
fabD	BUM88_03885	F- TTGCAGAAGCTTTGGAACAAACT<br>R- CG**C**AATTGAGCAACATCGGTAGC	97 bp
rpoB	BUM88_01345	F- ACGCCTAAAGGTGAAACTCAGTTAA<br>R- GTACCAGATGGAACACGTAAAGATG	110 bp
rpoD	BUM88_15640	F- GTTGCTGAAGAAGAAGCTGCTG<br>R- ACTGTACCCATTTCACGCATGTA	101 bp

TABLE S5. Primer sequences of the proposed reference genes (and corresponding locus tag) for *Acinetobacter haemolyticus* strain HW-2A (NCBI Reference Sequence: NZ_CP030880.1). Nucleotides different from the primers of the *A. baumannii* ATCC 19606 are in red, bold and underlined.

Gene	Locus tag	Forward (F) and Reverse (R) primer sequences (5' > 3')	Amplicon size
fabD	DS737_RS12015	F- TTGC**T**GAAGCTTTGGAACAAACT<br>R- CG**C**AATTGAGCAACATC**A**GT**C**G	97 bp
rpoB	DS737_RS14580	F- AC**A**CCTAAAGGTGAAACTCAGTTAA<br>R- GTACCAGA**C**GGAACACGTAAAGATG	110 bp
rpoD	DS737_RS03780	F- GTTGCTGAAGAAGAAGCTGCTG<br>R- AC**C**GTACCCATTTCACGCATGTA	101 bp

TABLE S6. Primer sequences of the proposed reference genes (and corresponding locus tag) for *Acinetobacter johnsonii* strain M19 (NCBI Reference Sequence: NZ_CP037424.1). Nucleotides different from the primers of the *A. baumannii* ATCC 19606 are in red, bold and underlined.

Gene	Locus tag	Forward (F) and Reverse (R) primer sequences (5' > 3')	Amplicon size
fabD	E0Z08_RS14520	F- T**C**GC**GC**AAGCTTTGGAACAAACT<br>R- CG**G**A**C**TTG**T**GC**T**ACATC**T**GTAGC	97 bp
rpoB	E0Z08_RS01475	F- ACGCCTAAAGGTGAAAC**G**CAGTTAA<br>R- GTACC**T**GATGGAACACGTAAAGATG	110 bp
rpoD	E0Z08_RS14165	F- GTTGCTGAAGAAGAAGC**T**GCTG<br>R- AC**A**GTACCCATTTCACGCAT**A**TA	101 bp

TABLE S7. Primer sequences of the proposed reference genes (and corresponding locus tag) for *Acinetobacter junii* strain 65 (NCBI Reference Sequence: NZ_CP019041.1). Nucleotides different from the primers of the *A. baumannii* ATCC 19606 are in red, bold and underlined.

Gene	Locus tag	Forward (F) and Reverse (R) primer sequences (5’ > 3’)	Amplicon size
fabD	BVL33_RS05735	F- TTGC**T**GAAGCTTTGGAACAAACT<br>R- CG**C**AATTGAGCAACATC**T**GTCGC	97 bp
rpoB	BVL33_RS15980	F- AC**A**CCTAAAGGTGAAACTCAGTTAA<br>R- GT**G**CC**T**GACGGAACACGTAAAGATG	110 bp
rpoD	BVL33_RS13665	F- GTTGC**T**GAAGAAGAAGCTGCTG<br>R- ACTGTACCCATTTC**G**CGCAT**A**TA	101 bp

TABLE S8. Primer sequences of the proposed reference genes (and corresponding locus tag) for *Acinetobacter lwoffii* strain 12CE1 (GenBank: CP059081.1). Nucleotides different from the primers of the *A. baumannii* ATCC 19606 are in red, bold and underlined.

Gene	Locus tag	Forward (F) and Reverse (R) primer sequences (5’ > 3’)	Amplicon size
fabD	H0S56_11100	F- TTGC**TCCG**GC**A**TTGGAACAAAC**C**<br>R- CG**C**AATT**C**A**T**C**T**ACAT**T**GGT**T**GC	97 bp
rpoB	H0S56_13020	F- AC**C**CCTAAAGGTGAAAC**A**CA**AC**T**T**A<br>R- GTACC**T**GATGGAAC**G**CGTAAAGATG	110 bp
rpoD	H0S56_03915	F- GTTGC**A**GAAGAAGAAGCTGC**A**G<br>R- ACTGTACCCAT**C**TCACGCAT**A**TA	101 bp

TABLE S9. Primer sequences of the proposed reference genes (and corresponding locus tag) for *Acinetobacter nosocomialis* strain KAN02 (GenBank: CP036171.1). Nucleotides different from the primers of the *A. baumannii* ATCC 19606 are in red, bold and underlined.

Gene	Locus tag	Forward (F) and Reverse (R) primer sequences (5’ > 3’)	Amplicon size
fabD	KAN02_14800	F- TTGCAGAAGCTTTGGAACAAACT<br>R- CGTAATTGAGCAACATCGGTAGC	97 bp
rpoB	KAN02_17500	F- ACGCCTAAAGG**C**GAAACTCAGTTAA<br>R- GTACCAGATGGAACACGTAAAGATG	110 bp
rpoD	KAN02_04025	F- GTTGCTGAAGAAGAAGCTGCTG<br>R- ACTGTACCCATTTCACGCATGTA	101 bp

TABLE S10. Primer sequences of the proposed reference genes (and corresponding locus tag) for *Acinetobacter pittii* strain PHEA-2 (GenBank: CP002177.1). Nucleotides different from the primers of the *A. baumannii* ATCC 19606 are in red, bold and underlined.

**Gene**	**Locus tag**	**Forward (F) and Reverse (R) primer sequences (5' > 3')**	**Amplicon size**
fabD	BDGL_000087	F- TTGCAGAAGCTTTGGAACAAACT<br>R- CG**C**AATTGAGCAACATCGGTAGC	97 bp
rpoB	BDGL_003193	F- ACGCCTAAAGGTGAAACTCAGTTAA<br>R- GTACCAGATGGAACACGTAAAGATG	110 bp
rpoD	BDGL_002162	F- GTTGCTGAAGAAGAAGCTGCTG<br>R- ACTGTACCCATTTCACGCATGTA	101 bp

TABLE S11. Primer sequences of the proposed reference genes (and corresponding locus tag) for *Acinetobacter schindleri* strain SGAir0122 (NCBI Reference Sequence: NZ_CP025618.2). Nucleotides different from the primers of the *A. baumannii* ATCC 19606 are in red, bold and underlined.

**Gene**	**Locus tag**	**Forward (F) and Reverse (R) primer sequences (5' > 3')**	**Amplicon size**
fabD	C0119_RS11750	F- TTGC**TACG**GCTTTGGAACAAACT<br>R- CGTAAT**GC**A**TTC**ACATC**T**GT**T**GC	97 bp
rpoB	C0119_RS13590	F- AC**A**CCTAAAGGTGAAACTCAGTTAA<br>R- GT**G**CC**C**GATGGAACACGTAAAGA**A**G	110 bp
rpoD	C0119_RS04480	F- GTTGC**A**GAAGAAGAAGCTGC**A**G<br>R- AC**A**GTACCCATTTCACGCAT**A**TA	101 bp
